# Sex differences in iron stores and associations of body iron with cardiovascular risk factors in the middle-aged general population

**DOI:** 10.3389/fendo.2026.1832141

**Published:** 2026-05-15

**Authors:** Wei Li, Mats Fredriksson, Dženeta Nezirević Dernroth, Hevi Mahmod, Xiao-Mei Mai, Carl Johan Östgren, Xi-Ming Yuan

**Affiliations:** 1 Clinical Department of Obstetrics and Gynecology in Linköping, Region Östergötland, Linköping, Sweden; 2 Department of Biomedical and Clinical Sciences, Linköping University, Linköping, Sweden; 3 Healthcare Research Strategy Department, Region Östergötland, Linköping, Sweden; 4 Department of Public Health and Nursing, Norwegian University of Science and Technology (NTNU), Trondheim, Norway; 5 Centre for Medical Image Science and Visualization (CMIV), Linköping University, Linköping, Sweden; 6 Department of Health, Medicine, and Caring Sciences, Linköping University, Linköping, Sweden; 7 Clinical Department of Occupational and Environmental Medicine, Department of Health, Medicine and Caring Sciences, Linköping University, Linköping, Sweden

**Keywords:** cardiovascular disease, ferritin, hyperferritinemia, metabolic syndrome, total body iron (TBI)

## Abstract

**Background:**

Men and women exhibit clear differences in both atherosclerotic cardiovascular disease and iron metabolism, although specific cardiovascular risk factors associated with body iron stores remain unclear. This study investigates if there are sex-specific differences in ferritin or total body iron (TBI) and their associations with cardiovascular risk factors and the metabolic syndrome.

**Methods:**

A total of 1,272 participants (640 men, 632 women) were randomly selected from the Swedish CArdiopulmonary Bioimage Study (SCAPIS). Iron-related biomarkers including ferritin, hemoglobin, soluble transferrin receptor, sTfR/log ferritin index, and TBI were analyzed in relation to conventional cardiovascular risk factors.

**Results:**

Men had higher iron stores than both premenopausal and postmenopausal women. They also demonstrated a more adverse cardiovascular risk profile with higher body mass index (BMI), a higher prevalence of hypertension, hyperlipidemia, diabetes, and prior myocardial infarction and a more atherogenic lipid profile than women. The prevalence of hyperferritinemia was 21.6% in men versus 12.0% in women (p < 0.01), whereas that of metabolic syndrome was 34.5% in men versus 24.6% in women (p < 0.01). In both sexes, BMI, alcohol consumption, LDL cholesterol, and triglycerides were associated with ferritin levels. Unlike men, women showed a significant association between age or hypertension and ferritin levels. Furthermore, ferritin, TBI, and hyperferritinemia were associated with metabolic syndrome in both sexes.

**Conclusions:**

This study highlights sex-specific differences in body iron stores, prevalence of cardiovascular disease comorbidity, and cardiovascular metabolic status. Hyperferritinemia or TBI was associated with metabolic syndrome in both sexes. In women, but not in men, age or hypertension was associated with ferritin levels. The findings underscore the importance of considering body iron markers when assessing cardiovascular risk in the middle-aged general population. It may help in developing personalized diagnostic and managing approaches for unhealthy cardiovascular metabolic status.

## 1 Introduction

The manifestation and progression of atherosclerotic diseases show significant sex differences (1). In the general population, a significantly higher prevalence of coronary artery disease (CAD) was observed in men than in women, although women with multiple cardiovascular risk factors showed a CAD prevalence similar to that of men (2). While the incidence of myocardial infarction is higher in men than in women, various risk factors show a stronger correlation with myocardial infarction in women than in men (3). The observed difference in myocardial infarction risk between the sexes cannot be adequately explained by recognized atherosclerotic risk factors (4). The extent to which the higher prevalence of CAD in men compared with women is due to differences in risk factor levels remains to be determined (5). In the Swedish CArdiopulmonary bioImage Study (SCAPIS), the prevalence of imaging-detected carotid and coronary atherosclerosis is greater in men, with women aged 65 exhibiting a prevalence level comparable with that of men who were 11 to 13 years younger (6). Although the pronounced sexual dimorphism observed in the incidence and complications of atherosclerosis is generally recognized, there is a lack of research that thoroughly examines sex difference in atherosclerotic risk factors and the mechanisms behind sex as a biological variable in atherosclerotic cardiovascular diseases (7).

As an alternative perspective on the observed sex difference in the incidence and mortality of atherosclerotic cardiovascular diseases, the iron hypothesis was introduced by Jerome Sullivan in 1981 (8). It has been revised that redox-active iron in plaque tissue may contribute to the pathogenesis of atherosclerosis (9, 10). Since then, the hypothesis has gained increasing recognition for its potential relevance to gender disparities in atherosclerosis. However, there is still limited research examining sex differences in atherosclerotic risk factors and their associations with iron biomarkers in general middle−aged populations.

To assess body iron stores and tissue iron, five markers have been used in this study. Hemoglobin (Hb) stores more than half of total body iron and is commonly used to detect iron deficiency. Ferritin reflects the iron stores in bone marrow and provides an estimate of total body iron (11). Elevated ferritin levels (>300 ng/ml in men, >200 ng/ml in women) indicate metabolic hyperferritinemia and is linked to cardiometabolic risks (12). The soluble transferrin receptor (sTfR) reflects functional iron status, and elevated sTfR levels indicate increased cellular iron demands (13). The sTfR-F index (sTfR/log ferritin) is an established diagnostic tool for evaluating body iron stores (14–16). Total body iron (TBI) expressed in milligrams per kilogram (mg/kg) provides a precise estimate of iron levels, even without anemia (14, 15). These markers, alongside atherosclerotic risk factors, present an opportunity to explore the relationships between body iron stores and cardiovascular health.

The primary research question in this study is whether there are sex-specific differences in body iron stores and atherosclerotic risk factors within the SCAPIS (Swedish CArdioPulmonary bioImage Study) cohort. Additionally, this study aimed to examine associations between atherosclerotic risk factors and ferritin—associations between hyperferritinemia or TBI and metabolic syndrome, in the total population, as well as separately in men and women.

## 2 Methodology

### 2.1 Study population

The Swedish CArdioPulmonary bioImage Study (SCAPIS) is a population-based, multicenter cohort study including individuals aged 50–64 years from the general Swedish population (n = 30,154) (17). The present study is based on the SCAPIS iron biomarker subcohort, which randomly included 1,272 participants (640 men and 632 women) from the Linköping site with the aim of investigating sex-specific differences in ferritin and total body iron (TBI), and their associations with cardiovascular risk factors and the metabolic syndrome. The sample size calculation was based on logarithm‐transformed ferritin values derived from the original dataset of our previous study (Sci Rep, 2020;10(1):3838). A total sample size of 168 participants was estimated to provide 90% statistical power, with an alpha level of 0.05, to detect differences in ferritin levels between men and women. We further assumed an approximately even distribution of participants across ferritin categories (ferritin low, ferritin high, and hyperferritinemia). To ensure adequate statistical power for these subgroup analyses, a larger sample size was therefore included in the present study, as presented.

The following variables related to clinical characteristics, socioeconomic factors, and lifestyle were obtained from detailed questionnaires on health and lifestyle of the SCAPIS study: sex, age, history of hypertension and hyperlipidemia, alcohol consumption, physical activity level, smoking status, level of education, financial strain, foreign-born, single household/living alone. Diabetes was defined as present based on self-report from questionnaires, fasting p-glucose ≥ 7.0 mmol/l or HbA1c ≥ 48 mmol/mol.

Metabolic syndrome was determined according to criteria by the American Heart Association (18). The participants were classified as having metabolic syndrome or not, based on the presence of any three out of the following five parameters: elevated waist circumference (⩾88 cm in women; ⩾102 cm in men), elevated triglycerides (⩾1.7 mmol/l or drug treatment for elevated triglycerides), reduced HDL cholesterol (<1.3 mmol/l in women; <1.03 mmol/l in men), treatment with statins, elevated blood pressure (SBP ⩾130 or DBP ⩾85), treatment with anti-hypertensive drugs, elevated fasting glucose (⩾5.6 mmol/l), or treatment with antidiabetic drugs or insulin.

The mean age of the participants was 57 years, with a significant proportion in marital unions. A notable proportion lived alone (15.8%) and a small proportion were foreign-born (9%).

### 2.2 Body iron stores

Fasting blood samples were collected from the individuals included. EDTA-plasma were stored frozen until analysis and subsequently used to measure ferritin and sTfR, two critical indicators of iron metabolism and status. The assays for ferritin and sTfR were conducted in an accredited laboratory (ISO/IEC 17025; Diagnostikcentrum i Östergötland, Linköping, Sweden).

Ferritin was analyzed using a sandwich immunoassay method with ruthenium labeling (11). The chemiluminescent emission resulting from the assay was measured at a wavelength of 620 nm using the Cobas e602 system (Roche Diagnostics Scandinavia AB). sTfR analysis was performed using an immunoturbidimetric assay, which involved the detection of precipitated turbidity photometrically at dual wavelengths of 800 and 570 nm using the Cobas c 502 system. Ferritin levels at or below the median were classified as ferritin low, whereas levels above the median were classified as ferritin high. Median values specific to the total population, men, or women were applied accordingly.

To further characterize body iron stores, we calculated the sTfR/log ferritin index (sTfR-F index), a diagnostic tool utilized to evaluate body iron status (19, 20). This index was derived from the ratio of sTfR concentration to the logarithm of ferritin concentration. Additionally, TBI was computed from the ratio of sTfR to ferritin, expressed in milligrams per kilogram (mg/kg). The formula used for this calculation was as follows: body iron (mg/kg) = − [log (sTfR/F ratio) − 2.8229]/0.1207. This measurement provided a quantitative assessment of the total amount of iron stored in tissues (14, 21).

### 2.3 Laboratory assessment

In addition to body iron biomarkers, we evaluated various laboratory characteristics that are related to cardiometabolic health. These included lipid profiles, glucose levels, HbA1c, high-sensitivity c-reactive protein (hsCRP), and Hb. All laboratory analyses were conducted through routine clinical procedures in the same accredited laboratory (Diagnostikcentrum i Östergötland, Linköping, Sweden).

The lipid profile was assessed by measuring levels of total cholesterol, low-density lipoprotein (LDL), high-density lipoprotein (HDL), triglycerides (TG), apolipoprotein A1 (ApoA1), and apolipoprotein B (ApoB). Fasting glucose and HbA1c were used to assess glycemic control. hsCRP served as a marker of inflammation.

### 2.4 Statistical analysis

Continuous variables are presented as mean [standard deviation (SD)] or median [interquartile range (IQR)] as appropriate. Categorical variables are presented as counts and percentages. Comparisons between men and women were performed using χ2 tests for categorical variables, Student’s t-test for normally distributed continuous variables, and the Mann–Whitney U test for non-normally distributed continuous variables.

The association between cardiometabolic parameters (independent variable) and ferritin (high vs. low level, dependent variable) was examined by logistic regression analysis with backward stepwise adjustment. The covariates include sociodemographic data, alcohol consumption, smoking status, body mass index (BMI), physical activity level, hypertension, dyslipidemia, diabetes, and various laboratory variables. The results were presented as odds ratios (OR) with 95% confidence intervals (CI). The sex of the participants was defined according to the individual’s Swedish personal identity number. The association between ferritin, hyperferritinemia, or TBI (independent variable) and metabolic syndrome (dependent variable) was examined by logistic regression analysis with multiple levels of adjustments. OR with 95% CI were reported. In model 1, ferritin or hyperferritinemia or TBI was incorporated. In model 2, sex and age were added as covariates. In model 3, the following variables were added to model 2: BMI, current smoking, alcohol consumption, sedentary (accelerometry measurement), moderate and vigorous-intensity physical activity (accelerometry measurement), and stress. In model 4, the following laboratory variables were added to model 3: hsCRP, total cholesterol, and LDL.

To assess the relative importance of risk factors in men and women, sex-specific logistic regression analyses were performed by using the same adjustment model as above except the sex.

All statistical tests were two-tailed, and a p-value of less than 0.05 was considered statistically significant. All statistical analyses were performed using IBM SPSS statistics 28 (IBM, Armonk, NY, USA) and GraphPad Prism (version 9.1.2).

## 3 Results

### 3.1 Descriptive characteristics

Clinical parameters, socioeconomic factors, and lifestyle factors were compared between men and women in low and high ferritin subgroups based on their respective median ferritin values (**Table 1**). In both low and high ferritin groups, men had significantly higher BMI, larger waist circumference, greater alcohol consumption, and higher systolic blood pressure and were more sedentary compared with women.

**Table 1:** Characteristics of study participants according to ferritin levels.

Variables	Ferritin low			Ferritin high		
	Total	Men	Women	Total	Men	Women
	n = 636	n = 320	n = 316	n = 636	n = 320	n = 316
Sociodemographic data						
Ages, mean (SD) year	56.9 (4.5)	**57.6 (4.6)**	**56.3 (4.4)**	57.8 (4.3)	**57.2 (4.3)**	**58.4 (4.1)**
Married	475 (74.7)	238 (74.4)	237 (70.7)	498 (78.3)	251 (78.4)	247 (83.4)
Living alone	113 (17.7)	56 (17.4)	57 (17.0)	87 (13.6)	**39 (8.5)**	**48 (16.2)**
Foreign born	54 (8.5)	34 (10.6)	20 (6.3)	39 (6.1)	17 (5.3)	19 (6.0)
Anthropometry						
BMI, mean (SD) kg/m2	26.3 (4.6)	**26.8 (4.4)**	**25.7 (4.7)**	27.7 (4.5)	**28.1 (4.3)**	**27.3 (4.8)**
Waist, mean (SD) cm	90.7 (13.4)	**96.4 (12.0)**	**88.8 (12.2)**	95.2 (13.1)	**100.3 (11.8)**	**90.1 (12.4)**
Smoking	58 (9.1)	28 (8.7)	30 (9.5)	59 (9.3)	26 (8.1)	33 (10.4)
Alcohol consumption						
≤ once/month	173 (27.2)	**70 (21.9)**	**103 (32.6)**	107 (16.8)	45 (14.1)	62 (19.6)
2–4 times/month	252 (39.6)	124 (38.7)	128 (40.5)	239 (37.6)	**107 (33.4)**	**132 (41.8)**
> once/week	199 (31.3)	**119 (37.2)**	**80 (25.3)**	272 (42.8)	**157 (49.1)**	**115 (36.4)**
Socioeconomic data						
High education	276 (43.4)	**123 (38.4)**	**153 (48.1)**	243 (38.2)	122 (38.1)	121 (38.3)
Employed	547 (86.0)	278 (86.9)	269 (85.1)	562 (88.4)	285 (89.1)	277 (87.7)
Perception of constant stress last year	121 (19.0)	52 (16.2)	69 (21.8)	108 (17.0)	52 (16.2)	56 (17.7)
No difficulties managing regular expenses, last 12 months	611 (96.1)	307 (95.9)	304 (96.2)	621 (97.6)	314 (98.1)	307 (97.1)
Ability to find 20,000 SEK in a week for unforeseen events	582 (91.7)	294 (91.9)	288 (91.1)	594 (93.5)	300 (93.7)	294 (93.0)
Physical activity						
Moderate-and vigorous intensity PA^a^, median (IQR)	6 (4 – 8)	6 (4 – 8)	6 (4 – 8)	5 (3 – 7)	6 (4 – 8)	6 (4 – 8)
Being sedentary^a^, median (IQR)	54 (47 – 61)	**57 (50 – 63)**	**52 (46 – 58)**	57 (50 – 63)	**58 (50 – 64)**	**53 (46 – 58)**
Medical history						
Hypertension	104 (16.3)	**67 (20.9)**	**37 (11.7)**	157 (24.7)	79 (24.7)	78 (24.7)
Hyperlipidemia	65 (10.2)	**41 (12.8)**	**24 (7.6)**	69 (10.8)	42 (13.1)	27 (8.5)
Diabetes	49 (7.7)	**32 (10.0)**	**17 (5.4)**	57 (8.9)	32 (10.0)	25 (7.9)
Previous stroke	10 (1.6)	8 (2.5)	2 (0.6)	4 (1.2)	2 (0.6)	2 (0.6)
Previous MI	16 (2.5)	**15 (4.7)**	**1 (0.3)**	6 (0.9)	4 (1.2)	2 (0.6)
Blood pressure						
- Systolic, mean (SD) mmHg	130.0 (17.3)	**130.9 (15.9)**	**101.5 (10.2)**	135.0 (17.6)	**135.2 (17.4)**	**130.8 (17.3)**
- Diastolic, mean (SD) mmHg	83.0 (10.8)	82.7 (10.6)	62.4 (9.7)	86.3 (11.1)	85.3 (10.8)	84.5 (10.4)
Clinical chemistry						
Total cholesterol, mean (SD) mmol/L	5.2 (1.0)	**5.0 (1.0)**	**5.4 (0.9)**	5.5 (1.1)	**5.4 (1.1)**	**5.6 (1.1)**
LDL, mean (SD), mmol/L	3.1 (0.9)	3.1 (0.9)	3.1 (0.8)	3.3 (1.0)	3.4 (1.0)	3.3 (1.0)
HDL, mean (SD) mmol/L	1.6 (0.4)	**1.4 (0.4)**	**1.8 (0.4)**	1.6 (0.5)	**1.4 (0.4)**	**1.8 (0.5)**
TG, mean (SD) mmol/L	1.1 (0.6)	**1.2 (0.7)**	**1.0 (0.5)**	1.4 (0.8)	**1.5 (1.0)**	**1.2 (0.7)**
ApoA1, mean (SD) g/L	1.6 (0.3)	**1.5 (0.2)**	**1.7 (0.3)**	1.5 (0.3)	**1.4 (0.2)**	**1.7 (0.3)**
ApoB, mean (SD) g/L	0.9 (0.2)	0.9 (0.2)	0.9 (0.2)	1.0 (0.2)	**1.0 (0.2)**	**1.0 (0.2)**
HbA1c, mean (SD) mmol/mol	36.5 (7.1)	37.0 (7.9)	36.1 (6.0)	36.8 (8.1)	36.9 (9.7)	36.7 (6.1)
Glucose, mean (SD) mmol/L	5.7 (1.2)	**5.9 (1.4)**	**5.5 (1.0)**	5.9 (1.3)	**6.1 (1.6)**	**5.8 (0.9)**
hsCRP, mean (SD) mg/L	1.7 (3.3)	2.0 (4.3)	1.5 (1.9)	2.5 (6.1)	2.5 (6.6)	2.5 (5.5)

Data are presented as n (%) unless otherwise indicated.

ApoA1, apolipoprotein A1; ApoB, apolipoprotein B; BMI, body mass index; Hb, hemoglobin; Hba1c, hemoglobin A1c; HDL, high-density lipoprotein; hsCRP, high-sensitivity c-reactive protein; LDL, low-density lipoprotein; MI, myocardial infarction; TG, triglyceride.

Participants were divided into two groups according to ferritin median levels as ferritin low (ferritin </= median value) or ferritin high (ferritin > median value). The ferritin median for the total population, men, or women was used according to their own median values. Statistical comparisons were made between men and women in each group. Data in bold indicate statistically significant differences between men and women, p < 0.05.

a Accelerometry measurement.

Notably, in the low ferritin group, men had a significantly higher prevalence of hypertension, hyperlipidemia, diabetes, and previous myocardial infarction than women. In this group, men were older than their female counterparts; conversely, in the group with high ferritin levels, women were older than men (**Table 1**). Results from clinical biochemistry analysis showed differences in lipid profile and glucose between sexes, with men generally having higher glucose and triglyceride values, but lower levels of total cholesterol, HDL, and ApoA1, regardless of ferritin level category. The levels of LDL, HbA1c, and sCRP were similar between sexes (**Table 1**).

### 3.2 Sex differences in body iron store

There were significant differences in body iron stores between men and women, as shown in **Table 2** and **Supplementary Table 1**. Men had significantly higher levels of body iron stores than women: ferritin 222.7 vs. 110.7 µg/L; TBI 13.8 vs. 11.0 mg/kg; Hb 149.9 vs. 136.3 g/L, whereas sTfR/F index 1.18 vs. 1.56 (**Table 2**). Among the participants, only one woman showed low hemoglobin levels, decreased ferritin, and elevated sTfR levels, indicating potential iron deficiency.

**Table 2:** Sex difference in body iron stores.

Variables	Total	Men	Women	p
All data	n = 1272	n = 640	n = 632	
Ferritin (µg/L)	167.1 (141.4)	222.7 (154.4)	110.7 (99.1)	<0.0001
sTfR (mg/L)	2.63 (1.0)	2.59 (0.6)	2.67 (1.3)	ns
sTfR/F index	1.37 (1.8)	1.18 (0.4)	1.56 (2.6)	<0.001
TBI (mg/kg)	12.43 (3.4)	13.82 (2.7)	11.03 (3.4)	<0.0001
Hb (g/L)	143.2 (11.7)	149.9 (9.6)	136.3 (9.4)	<0.0001
Without hsCRP >10 (mg/L)	n = 1242	n = 621	n = 621	
Ferritin (µg/L)	165.9 (141.0)	221.7 (153.9)	110.2 (99.4)	<0.0001
sTfR (mg/L)	2.62 (1.0)	2.57 (0.6)	2.67 (1.3)	ns
sTfR/F index	1.37 (1.9)	1.18 (0.4)	1.57 (2.6)	<0.001
TBI (mg/kg)	12.41 (3.4)	13.82 (2.7)	11.01 (3.4)	<0.0001
Hb (g/L)	143.2 (11.6)	150.1 (9.5)	136.3 (9.3)	<0.0001

Values are mean (SD). Data were analyzed using the Student’s t-test and the Mann–Whitney test, yielding similar results in the comparison between men and women. Hb, hemoglobin; hsCRP, high-sensitivity C-reactive protein; TBI, total body iron; sTfR, soluble transferrin receptor; sTfR/F index, sTfR/log ferritin index.

To eliminate the impact of inflammation on sex differences in body iron store, we excluded participants with hsCRP levels exceeding 10 mg/L, which did not alter the sex differences (**Table 2**).

Since female participants included both pre-menopausal women (with menstruation in the last 12 months) and post-menopausal women (without menstruation in the last 12 months), we further examined differences in body iron stores among men, pre-menopausal women, and post-menopausal women, as summarized in **Supplementary Table 1**. Significant differences were found among the groups, with men having higher ferritin and body iron levels compared with both pre-menopausal and post-menopausal women. Furthermore, body iron store was significantly higher in post-menopausal women compared with pre-menopausal women (**Supplementary Table 1**).

### 3.3 Associations between atherosclerotic risk factors and ferritin

To explore the association between conventional atherosclerotic risk factors and ferritin levels (high vs. low level), we conducted a multivariable logistic regression analysis. As shown in **Table 3**, BMI and alcohol consumption were positively associated with ferritin levels, in both sexes. Unlike their male counterparts, women exhibited a significant association between age or hypertension and ferritin levels. In men, a positive relationship was observed between hypertension and ferritin without statistical significance. The positive correlation between overweight or obesity and ferritin levels as well as TBI levels is further illustrated in **Supplementary Figure 1**, which indicates that ferritin and TBI levels tend to increase with rising BMI in both sexes. Furthermore, we found positive associations between laboratory atherosclerotic risk factors (LDL and TG) and ferritin levels across the entire population without sex differences (**Table 3**). In addition, there was a positive correlation between hsCRP and ferritin in the entire population, but not in men or women separately (**Table 3**).

**Table 3:** Atherosclerotic risk factors associated with ferritin levels.

Variables	Total (n = 1221)		Men (n = 606)		Women (n = 615)	
	Unadjusted OR (95% CI)	Adjusted OR (95% CI)	Unadjusted OR (95% CI)	Adjusted OR (95% CI)	Unadjusted OR (95% CI)	Adjusted OR (95% CI)
Anthropometric and clinical variables						
Ages (per 1 year increase)	1.03 (1.0, 1.1)*	1.03 (1.0, 1.1)*	0.98 (0.9, 1.0)		1.09 (1.0, 1.1)**	1.09 (1.0, 1.1)**
BMI (1 kg/m^2^ increase)	1.05 (1.0, 1.1)**	1.05 (1.0, 1.1)**	1.05 (1.0, 1.1)#	1.06 (1.0, 1.1)*	1.04 (1.0, 1.1)#	1.04 (1.0, 1.1)#
Smoking (yes vs. no)	0.92 (0.6, 1.4)		0.94 (0.5, 1.8)		1.00 (0.5, 1.9)	
Alcohol consumption						
< 1 time /month	1.00 (reference)		1.00 (reference)		1.00 (reference)	
2–4 times/month	1.57 (1.1, 2.2)**	1.54 (1.1, 2.1)**	1.20 (0.7, 2.0)	1.19 (0.7, 1.9)	1.82 (1.2, 2.8)**	1.81 (1.0, 1.1)**
> 1 time/week	2.51 (1.8, 3.5)**	2.43 (1.7, 3.4)**	2.05 (1.2, 3.3)**	2.01 (1.2, 3.2)**	2.76 (1.7, 4.5)**	2.79 (1.7, 4.5)**
Hypertension (yes vs. no)	1.49 (1.1, 2.1)*	1.59 (1.0, 1.9)*	1.35 (0.8, 2.2)		1.97 (1.2, 3.2)**	1.83 (1.1, 2.9)*
Hyperlipidemia (yes vs. no)	0.76 (0.5, 1.2)		0.78 (0.4, 1.4)		0.71 (0.3, 1.5)	
Diabetes (yes vs. no)	0.79 (0.4, 1.6)		0.80 (0.3, 2.0)		0.78 (0.3, 2.3)	
Clinical chemistry						
Cholesterol (mmol/L)	0.48 (0.0, 5.1)		0.63 (0.0, 21.7)		0.18 (0.0, 4.7)	
LDL (mmol/L)	2.68 (0.3, 28.5)	1.33 (1.1, 1.5)**	2.19 (0.1, 76.9)	1.38 (1.3, 2.5)**	6.69 (0.2, 178)	1.22 (1.0, 1.5)#
HDL (mmol/L)	1.89 (0.2, 20.4)		1.16 (0.0, 41.6)		5.49 (0.2, 146)	
TG (mmol/L)	2.38 (0.8, 7.1)	1.71 (1.3, 2.2)**	2.20 (0.4, 11.5)	1.83 (1.3, 2.5)**	3.08 (0.7, 14.0)	1.42 (1.1, 2.0)#
Glucose (mmol/L)	1.19 (1.0, 1.4)*	1.17 (1.0, 1.4)#	1.19 (1.0, 1.5)		1.30 (1.0, 1.8)#	1.26 (1.0, 1.7)#
Hba1c (mmol/mol)	0.97 (0.9, 1.0)*	0.96 (0.9, 1.0)*	0.96 (0.9, 1.0)#		0.97 (0.9, 1.0)	
CRP (mg/L)	1.03 (1.0, 1.1)#	1.03 (1.0, 1.1)*	1.02 (1.0, 1.1)		1.06 (1.0, 1.1)	
Physical activity						
mvpa	1.00 (1.0, 1.0)		1.00 (1.0, 1.0)		1.00 (1.0, 1.0)	
Sed	1.00 (1.0, 1.0)		1.00 (1.0, 1.0)		1.00 (1.0, 1.0)	

BMI, body mass index; CI, confidence interval; OR, odds ratio. CRP, c-reactive protein; Hb, hemoglobin; Hba1c, hemoglobin A1c; HDL, high-density lipoprotein; LDL, low-density lipoprotein; TG, triglycerides. Mvpa, moderate-and vigorous intensity PA; Sed, being sedentary.

Crude and adjusted odds ratios for atherosclerotic risk factors according to ferritin levels, namely, ferritin low (ferritin </= median value) or ferritin high (ferritin > median value). Ferritin median for total, men, or women was used according to their own median values. The high-level group was compared with the low-level group.

Data are analyzed by multivariable logistic regression analysis by stepwise adjustment. *P< 0.05, **p< 0.01, #p<0.1.

### 3.4 Higher prevalence of metabolic syndrome and hyperferritinemia in men

**Figure 1:**
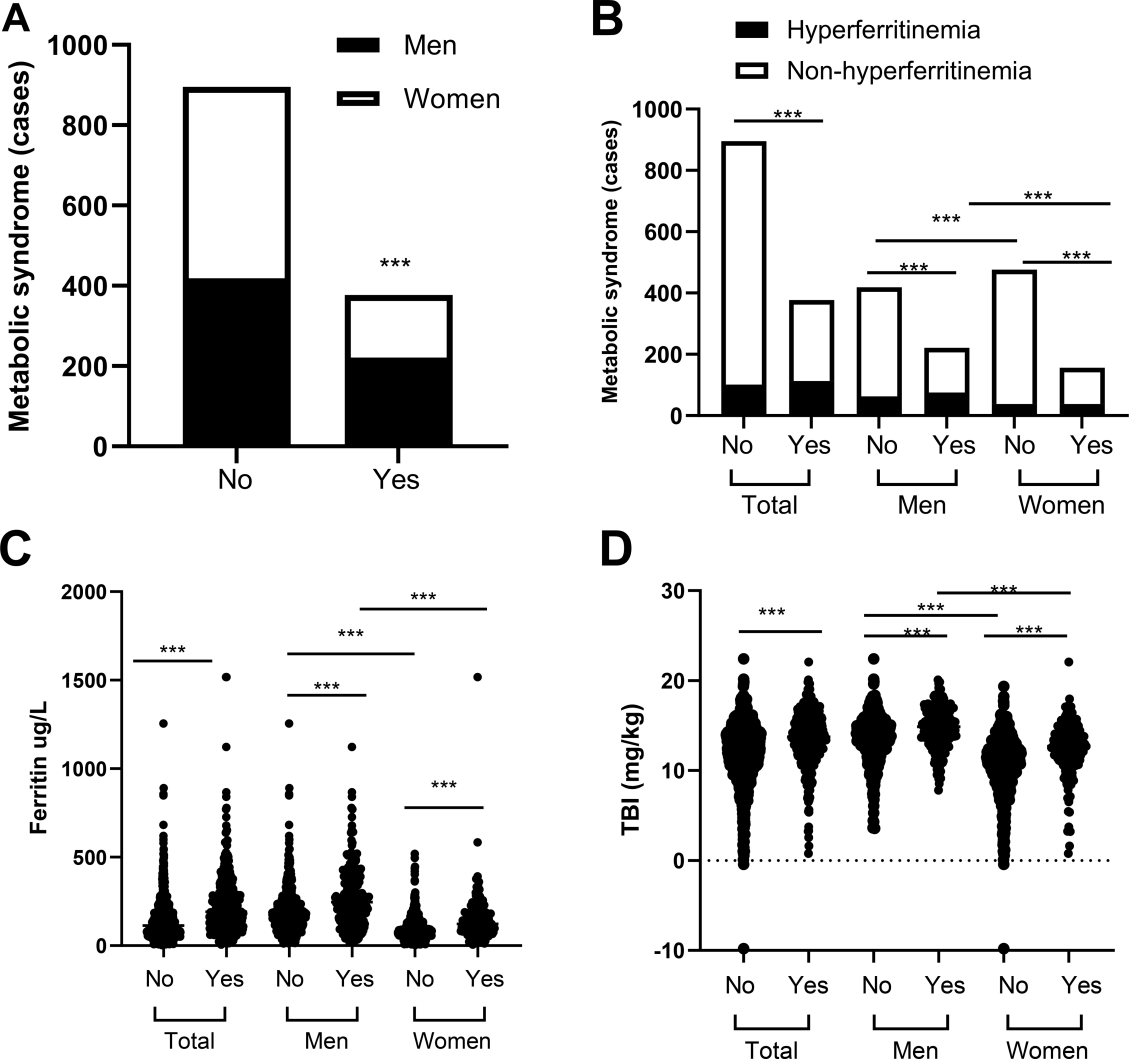
Men participants have higher numbers of metabolic syndrome and higher numbers of metabohyperferritinemia. **(A)** Significant higher numbers of metabolic syndrome in men, n of metabolic syndrome/non-metabolic syndrome = 221/419 for men and n = 156/476 for women. ***p < 0.001. **(B)** Men have significant higher numbers of hyperferritinemia in metabolic syndrome group. Hyperferritinemia/non-hyperfferitinemia in metabolic syndrome group n = 75/146 and for men and n = 38/118 for women; hyperferritinemia/non-hyperfferitinemia in non-metabolic syndrome group n = 63/356 for men and n = 38/438 for women. ***p < 0.001. **(C, D)** Significant higher levels of ferritin **(C)** and TBI **(D)** in participants with metabolic syndrome (Yes) as compared with metabolic healthy individuals (No). Data are mean ± SD. ***p < 0.001.

To study the relationship between iron metabolism and metabolic health status, we examined if there was an association of hyperferritinemia, ferritin, or TBI with metabolic syndrome. Metabolic syndrome was more prevalent in men than in women (**Figure 1A**). Participants with hyperferritinemia showed a significantly higher prevalence of metabolic syndrome compared with those without hyperferritinemia (**Figure 1B**). Further analysis revealed that individuals with metabolic syndrome had elevated levels of both ferritin and TBI in men and women (**Figures 1C, D**).

Since metabolic hyperferritinemia has been linked to an increased risk of cardiometabolic disease, we further examined the relationship between atherosclerotic risk factors and hyperferritinemia (**Supplementary Table 2**). Hyperferritinemia was observed in 16.8% of the study population (n = 214), with a significantly higher proportion of male participants (64.5%, p < 0.001). Individuals with hyperferritinemia exhibited significantly adverse cardiometabolic profiles, including elevated BMI, increased waist circumference, and higher systolic and diastolic blood pressure. They also had a greater prevalence of hypertension, hyperlipidemia, and diabetes. Moreover, these individuals had a more sedentary lifestyle and presented an atherogenic lipid profile. Their levels of glucose and hsCRP were also significantly higher compared with those without hyperferritinemia (**Supplementary Table 2**).

We subsequently investigated the variables for sex-specific differences, as detailed in **Supplementary Table 3**. In both hyperferritinemia and non-hyperferritinemia groups, sex-specific differences were noted including waist circumference, alcohol consumption, physical inactivity, systolic blood pressure, HDL, ApoA1, and hemoglobin concentrations. Notably, participants without hyperferritinemia displayed significant sex differences in BMI, hypertension, hyperlipidemia, history of myocardial infarction, total cholesterol, TG, and glucose levels. However, within the hyperferritinemia group, the only significant sex difference identified was in age, with women being older than their male counterparts (**Supplementary Table 3**). Moreover, the correlations between ferritin and ages (r = 0.25, p < 0.001) as well as TBI and ages (r = 0.21, p < 0.001) were found in women, but not in men (r = −0.49 for ferritin and ages and r = −0.02 for TBI and ages, p > 0.05).

### 3.5 Associations between ferritin, TBI, or hyperferritinemia and metabolic syndrome

To investigate the relationship between levels of ferritin, TBI, or hyperferritinemia and the presence of metabolic syndrome, we performed a multivariable logistic regression analysis. This analysis was adjusted for several covariates, including age, BMI, current smoking, alcohol consumption, physical activity, stress, hsCRP, total cholesterol, and LDL. The findings revealed a clear positive correlation between levels of ferritin or TBI and the incidence of metabolic syndrome in the entire population, including both male and female participants, even after adjustment (**Table 4**). Additionally, hyperferritinemia showed a significant association with metabolic syndrome in the overall cohort, as well as in both sexes, even after adjustment for multiple cardiovascular risk factors including inflammation marker hsCRP (**Table 4**).

**Table 4:** Associations of ferritin and hyperferritinemia with metabolic syndrome.

Variables	Total OR (95% CI)	p	Men OR (95% CI)	p	Women OR (95% CI)	p
Ferritin						
Model 1	2.97 (2.3, 3.8)	<0.001	2.54 (1.8, 3.6)	<0.001	3.79 (2.5, 5.7)	<0.001
Model 2	2.93 (2.3, 3.8)	<0.001	2.66 (1.9, 3.8)	<0.001	3.34 (2.2, 5.1)	<0.001
Model 3	3.00 (2.2, 4.2)	<0.001	2.85 (1.8, 4.5)	<0.001	3.71 (2.2, 6.2)	<0.001
Model 4	3.00 (2.1, 4.2)	<0.001	2.78 (1.8, 4.4)	<0.001	3.85 (2.3, 6.5)	<0.001
TBI						
Model 1	2.23 (1.7, 2.9)	<0.001	1.84 (1.3, 2.6)	<0.001	2.95 (2.0, 4.4)	<0.001
Model 2	2.16 (1.7, 2.8)	<0.001	1.85 (1.3, 2.6)	<0.001	2.62 (1.7, 3.9)	<0.001
Model 3	2.22 (1.6, 3.0)	<0.001	1.62 (1.1, 2.5)	<0.05	3.30 (2.0, 5.4)	<0.001
Model 4	2.20 (1.6, 3.0)	<0.001	1.56 (1.0, 2.4)	<0.05	3.33 (2.0, 5.6)	<0.001
Hyperferritinemia						
Model 1	3.43 (2.5, 4.6)	<0.001	3.08 (2.1, 4.5)	<0.001	3.53 (2.2, 5.8)	<0.001
Model 2	3.23 (2.4, 4.4)	<0.001	3.19 (2.1, 4.7)	<0.001	3.22 (1.9, 5.3)	<0.001
Model 3	3.01 (2.1, 4.4)	<0.001	2.77 (1.7, 4.5)	<0.001	3.24 (1.8, 6.0)	<0.001
Model 4	2.89 (2.0, 4.2)	<0.001	2.70 (1.6, 4.4)	<0.001	3.24 (1.7, 6.0)	<0.001

Associations of ferritin and hyperferritinemia with metabolic syndrome were analyzed using multivariable logistic regression models.

Model 1: without adjustment; model 2: adjusted by sex and ages; model 3: adjusted as model 2 + smoking, alcohol consumption, perception of constant stress last year, being sedentary, BMI; model 4: model 3 + total cholesterol, hsCRP, and LDL-cholesterol.

## 4 Discussion

In this study, we characterized sex differences in body iron stores in the form of five biomarkers, as well as atherosclerotic risk factors stratified by ferritin levels, and identified the relationship between ferritin and atherosclerotic risk factors. Several well-established atherosclerotic risk factors were associated with ferritin levels, including BMI, alcohol consumption, LDL, and TG in both sexes. Association of age and hypertension with ferritin was observed in women only. Furthermore, men had a higher prevalence of hyperferritinemia and metabolic syndrome than women. Moreover, levels of ferritin, TBI, and hyperferritinemia were associated with metabolic syndrome across sexes, even after adjustment for multiple cardiovascular risk factors. To our knowledge, this is the first study to examine associations between TBI expressed in milligrams per kilogram and metabolic syndrome in the middle-aged general population with adjustment of several cardiovascular risk factors and the inflammation marker hsCRP. We are also the first to identify several sex-based differences in cardiovascular risk factors in participants with ferritin levels under hyperferritinemia, such as BMI, hypertension, hyperlipidemia, previous myocardial infarction, total cholesterol, TG, and glucose. Recent research highlights that men exhibit a more adverse cardiovascular risk profile, particularly in terms of hypertension (22, 23), glucose levels (24, 25), and lipid metabolism (26). Our findings show that at different ferritin levels, men, compared with women, have higher systolic blood pressure, BMI, waist circumference, glucose, and greater prevalence of hypertension, hyperlipidemia, previous myocardial infarction, and increased regular alcohol consumption and sedentary behavior. Furthermore, alcohol consumption showed a positive association with ferritin levels in the total population and in both sexes. This may highlight the interplay between body iron stores and lifestyle factors, such as alcohol consumption.

Emerging evidence suggests that ferritin may serve as a biomarker for metabolic health and the onset and progression of metabolic disorders (12). Hyperferritinemia is frequently observed clinically in individuals with metabolic syndrome, and abnormalities in iron metabolism increase the risk of mortality in the general population (27), highlighting a potential link between body iron stores and cardiometabolic risk. Therefore, understanding the complex relationship between body iron stores and metabolic syndrome is vital for effectively managing the risk of atherosclerosis. In a recent paper, it was reported that lower serum iron levels were observed in patients with elevated BMI, metabolic syndrome, arterial hypertension, and type 2 diabetes, although inflammatory biomarkers involved in the iron homeostasis–inflammation axis such as soluble transferrin receptor were not available for analysis (28). In our study, we find that elevated plasma levels of ferritin or TBI and hyperferritinemia are associated with metabolic syndrome. The results suggest that hyperferritinemia linked to increased cardiometabolic risk may contribute to higher cardiovascular mortality, as reported in the Copenhagen City Heart Study, which showed that ferritin ≥ 200 µg/L is a predictor of early death in the general population (29). Since ferritin is an important marker for total body iron, our findings provide further support for the growing evidence linking iron metabolism and the pathophysiology of metabolic syndrome.

In contrast to most of the studies using serum ferritin, our study confirmed associations between plasma ferritin and several modifiable and non-modifiable cardiovascular risk factors including BMI, LDL, triglycerides, obesity, and alcohol consumption without major sex differences except age and hypertension for women only. The female−specific association between hypertension and ferritin offers meaningful vision into the underlying pathophysiology of hypertension and underscores the importance of sex−specific considerations in clinical management. The underlying mechanism is probably due to hormonal alteration during the menopause or post-menopause period that may simultaneously accelerate cardiovascular aging and increase cardiovascular risk as well as iron retention (30). Moreover, iron retention may in turn lead to oxidative stress and potentially contribute to cardiovascular damage. The explanation for the association of age with ferritin only in women could be due to a constant increase in iron stores in women throughout their life, especially between 40 and 60 years old, whereas in men, constant higher levels of iron status were found after 30 years of age (31). The present study extends the previous findings showing the sex-specific association of age with ferritin in women. The correlation of age with TBI in women is distinctive in the present study.

Although there are sex differences in iron markers, their interactions with hsCRP remain unclear. Given the impact of inflammation on iron biomarkers, it is important to assess whether the observed sex difference in iron markers is related to inflammation. After excluding individuals with hsCRP >10 mg/L, we still observed sex differences in iron biomarkers suggesting that the sex difference in iron metabolism is likely independent of inflammation. In addition, there is limited research on sex differences in the relationship between body iron stores and hsCRP. Our results show a slight positive correlation between ferritin and hsCRP in the entire population, suggesting that elevated ferritin may be related to inflammation in the general population.

There are several limitations to consider in this study. The cross-sectional design restricts the ability to establish causality from the observed associations. Despite adjustment for potential confounding variables, there remains a possibility that these and other unmeasured variables may affect the results. While the sample size is sufficient for the analysis, expanding it in future studies could strengthen the findings. The causal association of ferritin or TBI levels and hyperferritinemia with cardiovascular morbidity needs wider population-based RCTs to exclude confounding.

In conclusion, our study highlights sex differences in body iron stores and cardiovascular risk factors in the SCAPIS cohort. There was a notable correlation between levels of ferritin or TBI and metabolic syndrome, with hyperferritinemia showing a significant association with metabolic syndrome in both men and women. The findings underscore the importance of considering body iron markers when assessing cardiovascular risk in the middle-aged general population. It may help in developing personalized diagnostic and managing approaches for both men and women with unhealthy cardiovascular metabolic status.

## Data Availability

The data supporting the conclusions of this article will be made available by the corresponding author upon reasonable request.
