# Supplementary material for: Sex differences in iron stores and associations of body iron with cardiovascular risk factors in the middle-aged general population
Source: Front Endocrinol (Lausanne). 2026 May 15;17:1832141. doi: 10.3389/fendo.2026.1832141 (PMC13218848; doi:10.3389/fendo.2026.1832141)
Supplement: Supplementary file 1 [file DataSheet1.pdf]

SUPPLEMENTAL MATERIAL

Sex Differences in Iron Stores and Associations of Body Iron with Cardiovascular Risk Factors in the Middle-Aged General Population

Wei Li[1,2*], Mats Fredriksson[2,3], Dženeta Nezirević Dernroth[2,3], Hevi Mahmod[1], Xiao-Mei Mai[4], Carl Johan Östgren[5,6], Xi-Ming Yuan[7]

[1]Clinical Department of Obstetrics and Gynecology in Linköping, Region Östergötland

[2]Department of Biomedical and Clinical Sciences, Linköping University, Linköping, Sweden.

[3]Healthcare Research Strategy Department, Region Östergötland, Linköping, Sweden

[4]Department of Public Health and Nursing, NTNU, Norwegian University of Science and Technology, Trondheim, Norway.

[5]Centre for Medical Image Science and Visualization (CMIV), Linköping University, Linköping, Sweden.

[6]Department of Health, Medicine, and Caring Sciences, Linköping University, Linköping, Sweden.

[7]Clinical Department of Occupational and Environmental Medicine, Department of Health, Medicine and Caring Sciences, Linköping University, Linköping, Sweden

Supplementary Table I. Difference in body iron stores in men, pre-menopausal and postmenopausal women.

Supplemental Table II. Characteristics of participants in total study population, in hyperferritinema and non-hyperferritinemia group.

Supplemental Table III. Characteristics of study participants according to hyperferritinemia.

Supplemental Figure I. Men participants have higher numbers of metabolic syndrome and higher numbers of metabohyperferritinemia.

Supplementary Table I. Difference in body iron stores in men, pre-menopausal and postmenopausal women.

	Men<br>n = 640	Women<br>n = 632	Women Pre-M<br>n = 112	Women Post-M<br>n = 500	p
Ferritin (µg/L)	222.7 (154.4)	110.7 (99.1)	71.3 (66.6)	119.8 (104)	**a, b, c, d**
sTfR (mg/L)	2.59 (0.6)	2.67 (1.3)	2.60 (0.7)	2.70 (1.4)	
sTfR/F index	1.18 (0.4)	1.56 (2.6)	1.70 (0.9)	1.54 (2.8)	**e, b, f**
TBI (mg/kg)	13.82 (2.7)	11.03 (3.4)	9.26 (3.8)	11.41 (3.1)	**a, b, c, d**
Hb (g/L)	149.9 (9.6)	136.3 (9.4)	134.4 (8.6)	136.8 (9.4)	**a, b, c, g**

Values are mean (SD). Data was analyzed by Kruskal-Wallis test for multiple group comparison and Mann-Whitney test was used for two group comparisons.

Hb – hemoglobin, TBI – total body iron, Pre-M – pre-menopausal, Post-M – post-menopausal, sTfR – soluble transferrin receptor, **s**TfR/F index – **s**TfR/log ferritin index.

[a]$p < 0.0001$ among groups. [b]$p < 0.0001$ men vs pre-M, [c]$p < 0.0001$ men vs post-M, [d]$p < 0.0001$ pre-M vs post-M, [e]$p < 0.01$ among groups, [f]$p < 0.01$ men vs post-M, [g]$p < 0.05$ pre-M vs post-M.

Supplementary Table II. Characteristics of participants in total study population, in hyperferritinema and non-hyperferritinemia group.

	**Total**<br>n = 1272	**Non-hyper-F**<br>n = 1058	**Hyper-F**<br>n = 214	**p**
**Sociodemographic data**				
Ages, year, mean (SD)	57.4 (4.4)	57.3 (4.4)	57.7 (4.5)	ns
Male (%)	640 (50.3)	502 (47.5)	138 (64.5)	<0.001
Married (%)	973 (76.5)	795 (75.1)	178 (83.2)	<0.05
Living alone (%)	201 (15.9)	177 (16.7)	24 (11.2)	ns
Foreign born (%)	90 (7.0)	73 (6.9)	17 (7.9)	ns
**Anthropometry**				
BMI (kg/m2), mean (SD)	27.0 (4.6)	26.7 (4.5)	28.6 (4.7)	<0.001
Waist, cm, mean (SD)	93.0 (13.4)	91.7 (13.0)	99.4 (13.6)	<0.001
**Smoking and alcohol habits**				
Smoking (%)	117 (9.2)	96 (9.1)	21 (9.8)	ns
Alcohol consumption (%)				
≤ once/month	280 (22.0)	245 (23.2)	35 (16.3)	<0.05
2-4 times/month	491 (38.6)	424 (40.1)	67 (31.3)	<0.05
> once/week	471 (37.0)	364 (34.4)	107 (35.2)	ns
**Socioeconomic data**				
High education (%)	522 (41.0)	434 (41.0)	88 (41.1)	ns
Employed (%)	1109 (87.2)	917 (86.7)	192 (89.7)	ns
Perception of constant stress last year (%)	239 (18.9)	205 (19.4)	34 (15.9)	ns
No difficulties managing regular expenses, last 12 months (%)	1201 (94.4)	999 (94.4)	202 (94.4)	ns
Ability to find 20000 SEK in a week for unforeseen events (%)	1179 (92.7)	976 (92.2)	200 (93.4)	ns
**Physical activity**				
Moderate-and vigorous intensity PA[a], median (IQR)	6 (4 – 8)	6 (4 – 8)	5 (3 – 7)	<0.001
Being sedentary[a], median (IQR)	55 (48 – 61)	54 (47 – 61)	57 (50 – 63)	<0.001
**Medical histroty**				
Hypertension (%)	261 (20.5)	200 (18.9)	61 (28.5)	<0.01
Hyperlipidemia (%)	131 (10.3)	100 (9.4)	31 (14.5)	<0.05
Diabetes (%)	106 (8.3)	75 (7.1)	31 (14.5)	<0.01
Previous stroke (%)	14 (1.1)	12 (1.1)	2 (0.9)	ns
Previous MI (%)	22 (1.7)	19 (1.8)	3 (1.4)	ns
**Blood pressure, mean (SD)**				

- Systolic (mmHg)	130.8 (17.4)	130.0 (17.3)	135.0 (17.6)	< 0.001
- Diastolic (mmHg)	83.5 (10.9)	83.0 (10.8)	86.3 (11.1)	< 0.001
**Clinical chemistry, mean (SD)**				
Total cholesterol (mmol/L)	5.32 (5.43)	5.35 (1.0)	5.50 (1.2)	ns
LDL (mmol/L)	3.21 (0.93)	3.18 (0.9)	3.35 (1.1)	<0.05
HDL (mmol/L)	1.61 (0.47)	1.65 (0.5)	1.43 (0.4)	<0.001
TG (mmol/L)	1.23 (0.75)	1.15 (0.6)	1.61 (1.1)	<0.001
ApoA1 (g/L)	1.56 (0.3)	1.58 (0.3)	1.49 (0.3)	<0.001
ApoB (g/L)	0.96 (0.2)	0.95 (0.2)	1.02 (0.3)	<0.001
Hb (g/L)	143.2 (11.7)	142.3 (11.6)	147.5 (11.2)	<0.001
HbA1c (mmol/mol)	36.7 (7.6)	36.4 (6.7)	37.9 (11.1)	ns
Glucose (mmol/L)	5.84 (1.26)	5.75 (1.1)	6.29 (1.7)	<0.001
hsCRP (mg/L)	2.13 (4.92)	1.97 (4.4)	2.89 (6.7)	<0.001

ApoA1 – apolipoprotein A1; ApoB – apolipoprotein B; BMI – body mass index; DBP – diastolic blood pressure; Hb – hemoglobin; Hba1c – hemoglobin A1c; HDL – high-density lipoprotein; hsCRP – high sensitive c-reactive protein; LDL – low-density lipoprotein; MI – myocardial infarction; SBP – systolic blood pressure; TG – triglyceride.

Statistical comparisons were made between men and women; data are presented as mean (SD) or percentages (%). [a]Accelerometry measurement.

Supplementary Table III. Characteristics of study participants according to hyperferritinemia

	Non-hyperferritinemia		Hyperferritinemia	
	Men (n = 502)	Women (n = 556)	Men (n = 138)	Women (n = 76)
**Sociodemographic data**				
Ages, mean (SD) year	57.4 (4.4)	57.2 (4.4)	57.2 (4.5)	58.7 (4.4)
Married	374 (74.5)	421 (75.7)	115 (83.3)	63 (82.9)
Living alone	82 (16.3)	95 (17.1)	14 (10.1)	10 (13.2)
Foreign born	41 (8.2)	32 (5.8)	10 (7.2)	7 (9.2)
**Anthropometry**				
BMI, mean (SD) kg/m2	**27.0 (4.1)**	**26.4 (4.9)**	29.1 (4.9)	27.8 (4.3)
Waist, mean (SD) cm	**97.1 (11.4)**	**86.8 (12.5)**	**103.1 (13.0)**	**92.6 (12.0)**
Smoking	43 (8.6)	53 (9.5)	11 (8.0)	10 (13.2)
Alcohol consumption				
≤ once/month	**98 (19.5)**	**147 (26.4)**	17 (12.3)	18 (23.67)
2-4 times/month	189 (37.7)	235 (42.1)	42 (30.4)	25 (32.9)
> once/week	**200 (39.8)**	**164 (29.5)**	**76 (55.1)**	**31 (40.8)**
**Socioeconomic data**				
High education	**187 (37.2)**	**247 (44.2)**	61 (44.2)	27 (35.5)
Employed	438 (87.2)	479 (86.1)	125 (90.6)	67 (88.2)
Perception of constant stress last year	91 (18.1)	114 (20.5)	18 (13.0)	16 (21.0)
No difficulties managing regular expenses, last 12 months	471 (93.8)	528 (95.0)	131 (94.9)	71 (93.2)
Ability to find 20000 SEK in a week for unforeseen events	463 (92.2)	513 (92.3)	131 (94.9)	69 (90.8)
**Physical activity**				
Moderate-and vigorous intensity PA[a], median (IQR)	6 (4 – 8)	6 (4 – 8)	5 (3 – 8)	5 (3 – 7)

Being sedentary[a], median (IQR)	**57 (50 – 63)**	**52 (46 – 58)**	**59 (51 – 64)**	**55 (47 – 61)**
**Medical history**				
Hypertension	**107 (21.3)**	**93 (16.7)**	39 (28.3)	22 (28.9)
Hyperlipidemia	**61 (12.1)**	**42 (8.3)**	22 (16.4)	9 (11.8)
Diabetes	43 (8.6)	32 (5.8)	21 (15.2)	10 (13.2)
Previous stroke	8 (1.6)	4 (0.7)	2 (1.5)	0 (0.0)
Previous MI	**17 (3.4)**	**2 (0.4)**	2 (1.5)	1 (1.3)
**Blood pressure**				
- Systolic, mean (SD) mmHg	**132.1 (16.6)**	**128.1 (17.6)**	**136.6 (17.0)**	**132.1 (18.4)**
- Diastolic, mean (SD) mmHg	83.2 (10.7)	82.7 (10.9)	86.7 (10.7)	85.6 (11.8)
**Clinical chemistry**				
Total cholesterol, mean (SD) mmol/L	**5.1 (1.0)**	**5.5 (1.0)**	5.5 (1.2)	5.5 (1.3)
LDL, mean (SD), mmol/L	3.2 (0.9)	3.2 (0.9)	3.3 (0.9)	3.2 (1.2)
HDL, mean (SD) mmol/L	**1.4 (0.4)**	**1.8 (0.4)**	**1.3 (0.3)**	**1.7 (0.5)**
TG, mean (SD) mmol/L	**1.3 (0.8)**	**1.1 (0.5)**	1.5 (1.0)	1.4 (0.8)
ApoA1, mean (SD) g/L	**1.5 (0.2)**	**1.7 (0.3)**	**1.4 (0.2)**	**1.6 (0.3)**
ApoB, mean (SD) g/L	0.9 (0.2)	0.9 (0.2)	**1.0 (0.3)**	**1.0 (0.3)**
Hb, mean (SD) g/L	**149.3 (9.6)**	**136 (9.4)**	**152.2 (9.3)**	**138.9 (9.0)**
HbA1c, mean (SD) mmol/mol	36.7 (7.8)	36.2 (5.5)	37.7 (11.9)	38.1 (9.4)
Glucose, mean (SD) mmol/L	**5.9 (1.3)**	**5.6 (0.9)**	6.4 (1.9)	6.1 (1.4)
hsCRP, mean (SD) mg/L	2.1 (5.1)	1.9 (3.7)	2.7 (6.9)	3.2 (6.3)

Values are presented as n (%) unless otherwise indicated. ApoA1 – apolipoprotein A1; ApoB – apolipoprotein B; BMI – body mass index; DBP – diastolic blood pressure; Hb – hemoglobin; Hba1c – hemoglobin A1c; HDL – high-density lipoprotein; hsCRP – high sensitive c-reactive protein; LDL – low-density lipoprotein; MI – myocardial infarction; SBP – systolic blood pressure; TG – triglyceride.

Participants were divided into two groups according to hyperferritinemia (ferritin > 300 µg/L for men and > 200 µg/L for women) or not. Statistical comparisons were made between men and women in each group; data are presented mean (SD) or percentages (%). Data in bold indicate statistically significant differences between men and women, $p < 0.05$. [a]Accelerometry measurement.

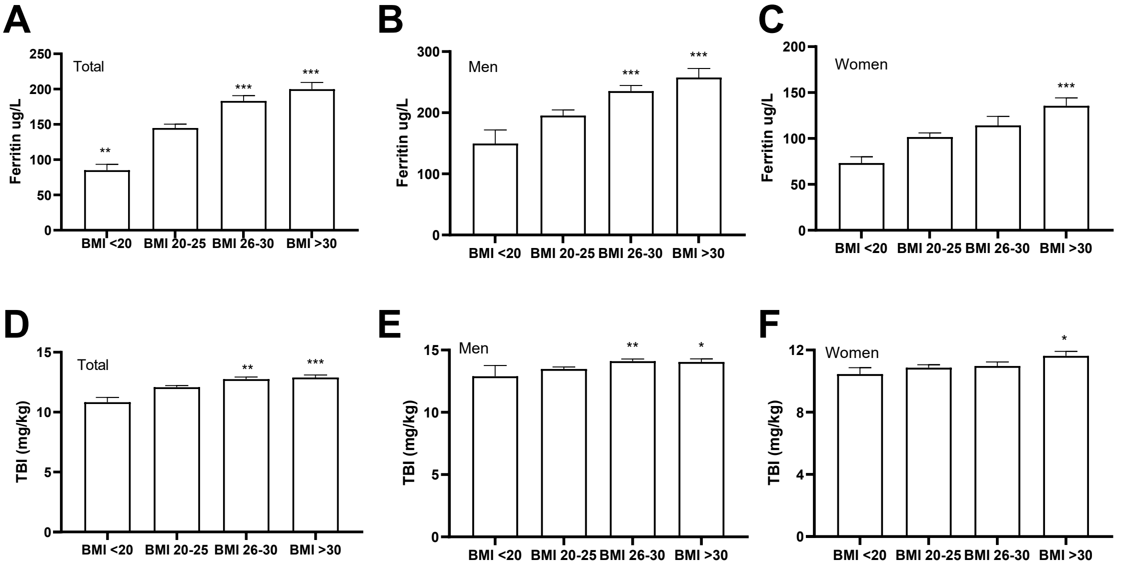

Supplementary Figure I. Significant higher levels of ferritin (A-C) and TBI (D-F) in participants with overweight and obesity. Data are mean ± SEM. *p<0.05, **P<0.01 and ***p<0.001 vs BMI 20 – 25.
